# Cell specific quantitative iron mapping on brain slices by immuno-µPIXE in healthy elderly and Parkinson’s disease

**DOI:** 10.1186/s40478-021-01145-2

**Published:** 2021-03-22

**Authors:** I. Friedrich, K. Reimann, S. Jankuhn, E. Kirilina, J. Stieler, M. Sonntag, J. Meijer, N. Weiskopf, T. Reinert, T. Arendt, M. Morawski

**Affiliations:** 1 Paul Flechsig Institute of Brain Research, Medical Faculty, Universität Leipzig, Leipzig, Germany; 2 Department of Neurophysics, Max Planck Institute for Human Cognitive and Brain Sciences, Leipzig, Germany; 3 Felix Bloch Institute for Solid State Physics, Faculty of Physics and Earth Sciences, Universität Leipzig, Leipzig, Germany; 4 Center for Cognitive Neuroscience Berlin, Freie Universität Berlin, Berlin, Germany

## Abstract

Iron is essential for neurons and glial cells, playing key roles in neurotransmitter synthesis, energy production and myelination. In contrast, high concentrations of free iron can be detrimental and contribute to neurodegeneration, through promotion of oxidative stress. Particularly in Parkinson’s disease (PD) changes in iron concentrations in the substantia nigra (SN) was suggested to play a key role in degeneration of dopaminergic neurons in nigrosome 1. However, the cellular iron pathways and the mechanisms of the pathogenic role of iron in PD are not well understood, mainly due to the lack of quantitative analytical techniques for iron quantification with subcellular resolution. Here, we quantified cellular iron concentrations and subcellular iron distributions in dopaminergic neurons and different types of glial cells in the SN both in brains of PD patients and in non-neurodegenerative control brains (Co). To this end, we combined spatially resolved quantitative element mapping using *m*icro *p*article *i*nduced *X*-ray *e*mission (µPIXE) with nickel-enhanced immunocytochemical detection of cell type-specific antigens allowing to allocate element-related signals to specific cell types. Distinct patterns of iron accumulation were observed across different cell populations. In the control (Co) SNc, oligodendroglial and astroglial cells hold the highest cellular iron concentration whereas in PD, the iron concentration was increased in most cell types in the substantia nigra except for astroglial cells and ferritin-positive oligodendroglial cells. While iron levels in astroglial cells remain unchanged, ferritin in oligodendroglial cells seems to be depleted by almost half in PD. The highest cellular iron levels in neurons were located in the cytoplasm, which might increase the source of non-chelated Fe^3+^, implicating a critical increase in the labile iron pool. Indeed, neuromelanin is characterised by a significantly higher loading of iron including most probable the occupancy of low-affinity iron binding sites. Quantitative trace element analysis is essential to characterise iron in oxidative processes in PD. The quantification of iron provides deeper insights into changes of cellular iron levels in PD and may contribute to the research in iron-chelating disease-modifying drugs.

## Introduction

Iron is essential for a proper CNS function. It plays an important role as cofactor of numerous enzymes and is involved in ATP production, myelination and synthesis of DNA, RNA, proteins, and neurotransmitters.

In the brain, variations in iron levels correlate with its structural integrity [42], and there is no other organ but the CNS that is in such a constant need for readily available iron [85]. Any mismatch in the demand and regional-temporal distribution of iron may result in neurological and/or mental dysfunction. Iron deficiency, for example, is a well-established cause for impaired motor and cognitive development [2, 70, 83, 95, 101]. On the other hand, increased levels of iron are harmful and iron accumulations are typical hallmarks of brain ageing and several neurodegenerative disorders particularly PD [34, 37, 38, 44, 72, 85, 91, 98]. Various factors have been suggested to account for increased iron accumulation in the SN of patients with PD such as, for example, dysfunction of the blood–brain barrier, altered cellular iron transport, an increased pro-inflammatory state and mutations in genes of iron transport, storage and binding [10, 13, 27, 53]. However, all these processes will differently affect different cell populations.

Still, the local accumulation of iron in the SN in patients with PD remains a controversial issue. Numerous studies on post mortem tissue report on an increased amount of total iron in the SN [22, 26, 51, 84] also supported by large body of in vivo findings from Magnetic Resonance Imaging (MRI) [9, 16, 46–48, 69, 82]. The increasing importance and approaches for in vivo brain iron assessment using multiparametric MRI is featured in a review by Moeller et al. [58]. Quantitative MRI may provide useful biomarkers for brain integrity assessment in iron-related neurodegeneration. Particularly, a prominent change in iron-sensitive T2* MRI contrast within the sub areas of the SN overlapping with nigrosome 1 were shown to be a hallmark of PD with high diagnostic power. Other studies failed to detect any disease-related differences [33, 49, 87]. Of note, more in-depth knowledge on whether changes in intracellular iron pools preferentially affect neurons or glial cells is limited by the lack of quantitative analytical techniques with sufficient spatial resolution for in-situ cellular analysis.

To glean much needed information about cellular and subcellular compartmentalisation of iron in health and disease, we have developed a powerful analytical approach, which we call immuno-*m*icro *p*article *i*nduced *X*-ray *e*mission (µPIXE). It allows for quantitative determining the iron concentrations of specific target structures in tissue sections with a spatial resolution in the µm range. To this end, quantitative element mapping using µPIXE was combined with nickel-enhanced immunocytochemical detection of cell type-specific antigens to specifically allocate element-related signals to specific cell types. In the present study, this method was applied to quantify the cell specific iron concentrations in SN nigrosome 1 in controls and PD.

## Materials and methods

### Tissue preparation

Midbrains comprising the nigrosome 1 of the SNc were obtained from individuals of both sexes with no signs of neuropathological alterations (8 cases; healthy controls, HC; mean age 66 ± 16 years) and from patients who died with a clinical diagnosis of idiopathic Parkinson’s disease (8 cases; PD; mean age 75 ± 7 years) (for detailed case profile see Table 1). The definite diagnosis of PD has been verified by neurohistopathological examination based on a severe loss of neuromelanin containing neurons in the nigrosome 1 of the SNc, the presence of extraneuronal melanin and Lewy bodies (Table 2). Although the appearance of Lewy bodies might be also a hallmark of dementia with Lewy bodies (DLB), a detailed synopsis of neuropathological examination and clinical presentation of the PD individuals included in our experimental analysis allowed a strict inclusion of patients that suffered exclusively from PD and did not present any comorbidity of dementia. Brains were fixed by immersion in 4% formaldehyde in 0.1% phosphate buffered saline (PBS, pH 7.4). Small blocks (25 mm × 25 mm) comprising the substantia nigra were dissected and embedded in Histowax following conventional protocols for paraffin embedding and block mounting. Subsequently, the tissue was sectioned (10 µm) and attached to microscope glass slides. Transverse sections throughout the midbrain were cut using a microtome (Jung Histoslide 2000, Leica).

**Table 1:** Case profile of the human subjects

	Case #	PMD (h)	Gender	Age (years)	Neuropathology (PD)	COD
CO	1	48	Female	78	control/ NAD	Respiratory failure
	2	26	Female	71	control/ NAD	Peritonitis
	3	24	Female	87	control/ NAD	Global insufficiency
	4	24	Male	75	control/ NAD	Bronchopneumonia
	5	24	Male	65	control/ NAD	Myocardial infarction
	6	51	Male	31	control/ NAD	Coronary failure
	7	48	Male	69	control/ NAD	Myocardial infarction
	8	72	Male	49	control/ NAD	Myocardial infarction
Mean		40 ± 16	3/5	66 ± 16		
PD	9	30	Female	67	clin./path	Myocardial infarction
	10	48	Female	77	clin./path	Bronchopneumonia
	11	48	Female	77	clin./path	Bronchopneumonia
	12	26	Male	82	clin./path	Renal failure
	13	26	Male	62	clin./path	Bronchopneumonia
	14	24	Male	72	clin./path	Myocardial infarction
	15	26	Male	88	clin./path	Pancreatitis
	16	34	Male	73	clin./path	Pulmonary embolism
Mean		33 ± 9	3/5	75 ± 7		

*PD* Parkinson’s disease, *PMD post mortem* delay, *COD* cause of death, *Clin* clinical diagnosis of PD, *path* pathological diagnosis of PD, *NAD* no abnormality detected

**Table 2:** Neuron count (mean ± SD) in the substantia nigra nigrosome 1

SN	Nissl^+^/mm^2^	TH^+^/mm^2^	Extraneuronal neuromelanin	Lewy bodies
Co	41 ± 12	14 ± 8	Negative	Negative
PD	15 ± 4	2.5 ± 1.5	Positive	Positive

*PD* Parkinson’s disease, *Co* Healthy control, *TH*+ Tyrosine hydroxylase

Brains were provided by the Brain Banking Centre Leipzig of the German Brain-Net (GZ 01GI9999-01GI0299), operated by the Paul Flechsig Institute of Brain Research. The entire procedure of case recruitment, acquisition of the patients’ personal data, the protocols and the informed consent forms, performing the autopsy and handling the autopsy material have been approved by the responsible authorities (Approval # 282-02 and Approval # 205/17-ek).

#### Identification of anatomical regions and applied nomenclature

Anatomical regions were identified on Nissl- and anti-HuCD-stained sections using the atlas of the human brain by Mai et al. [50]. Delineations and nomenclature of substructures of SN followed the detailed anatomical descriptions of the SN given by Braak and Braak [11], Fearnley and Lees [49], van Domburg and ten Donkelaar [88], McRitchie et al. [54] and Damier et al. [17, 18].

### Histochemistry

Histowax sections were deparaffinised, rehydrated in a descending alcohol series and incubated for 2 h at 37 °C in a mixture of 5% potassium ferrocyanide and 5% hydrochloric acid (Perls' stain). Subsequently, sections were rinsed in PBS (pH 7.4) and pre-incubated for 10 min in 0.5 mg/ml 3,3′-diaminobenzidine (DAB, Sigma) Tris–HCl. Subsequently, an incubation in DAB for 15 min at room temperature (0.5 mg DAB/ml Tris–HCl and 0.05% H_2_O_2_) was performed (DAB amplification of Perls’ stain). After further washing the sections successively in Tris–HCl (pH 8.0), PBS and purified water, sections were dehydrated, incubated in toluol and coverslipped with Entellan (Merck Millipore).

### Immunohistochemistry

For immunohistochemistry, sections were deparaffinised in xylene, rehydrated in a descending alcohol series and transferred to PBS (pH 7.4). To block endogenous peroxidase-activity, samples were incubated in 60% methanol containing 2% H_2_O_2_ (for 1 h at room temperature). Antigen retrieval pre-treatment prior to the staining process was performed as follows. Sections prepared for detection of CNP, IBA-1 and Olig2 were pre-treated with citric acid sodium citrate buffer (pH 6.0) and sections prepared for the detection of Hu C/D were pre-treated with Tris–HCl (pH 8.0), respectively for 20 min at 90 °C. After washing, sections were incubated overnight at room temperature in a solution (phosphate-buffered saline with 2% BSA, 0.3% milk powder and 0.5% donkey serum) containing the following primary antibodies: (1) mouse anti-human neuronal protein HuCD (1:400, Thermo Fisher Scientific) for neurons, (2) rabbit anti IBA-1 (1:800, Wako) for microglial cells, (3) rabbit anti-GFAP (1:500, Dako) for astroglial cells, (4) rabbit anti-Olig2 (1:100, Immuno Biological Laboratories) for oligodendroglial cells, (5) mouse anti-CNP (1:300, BioLegend) for oligodendroglial cells, (6) goat anti-ferritin heavy chain Y-16 (1:200, Santa Cruz Biotechnology) for ferritin and (7) rat anti-myelin basic protein (1:400, abcam) for myelin.

Subsequently, sections were rinsed in PBS-Tween (pH 7.4) and incubated in biotinylated secondary antibody solution (containing a mixture of PBS-T and phosphate-buffered saline with 2% BSA, 0.3% milk powder and 0.5% donkey serum, 2:1, for 1 h at room temperature) using donkey anti-mouse IgG, donkey anti-rat IgG, donkey anti-rabbit IgG and donkey anti-goat IgG (1:1000, Dianova, Germany). Binding sides were revealed by incubation for 1 h with peroxidase-labeled streptavidin (Extravidin®, 1:2000, Sigma Aldrich). Further, sections were rinsed in PBS-T and Tris–HCl (pH 8.0). Following treatment with peroxidase complex, the colour reaction was developed using DAB and nickel-ammonium sulphate (Sigma Aldrich, 99.999% purity grade). Finally, sections were rinsed in Tris–HCL and PBS-T, dehydrated in an ascending alcohol series, incubated in toluol and coverslipped with Entellan (Merck Millipore).

### Fluorescent immunohistochemistry

Histowax sections were deparaffinised, rehydrated and incubated in 60% methanol containing 2% H_2_O_2_ (for 1 h at room temperature), washed in PBS-T (pH 7.4) and incubated for 48 h at 4 °C in a solution (phosphate-buffered saline with 2% BSA, 0.3% milk powder and 0.5% donkey serum) with one of the following combinations of primary antibodies: (1) rabbit anti-IBA-1 (1:800, Wako) and goat anti-ferritin heavy chain Y-16 (1:200, Santa Cruz Biotechnology), (2) rabbit anti-GFAP (1:500, Dako) and goat anti-ferritin heavy chain Y-16 (1:200, Santa Cruz Biotechnology), (3) rabbit anti-Olig2 (1:100, Immuno Biological Laboratories) and goat anti-ferritin heavy chain Y-16 (1:200, Santa Cruz Biotechnology). Any pre-treatment of sections was performed corresponding to immunohistochemically procedures. Subsequently, brain sections were washed and incubated in a combination of fluorochrome-conjugated secondary antibodies (for 90 min at room temperature) using donkey anti-rabbit IgG (Dianova; Cy3, Cy5) and donkey anti-goat IgG (Dianova; Cy3, Cy5) for detection. Sections were washed and treated for 1 min with Sudan black B (Merck Millipore) dissolved in 70% EtOH to suppress auto-fluorescence and then differentiated in 70% EtOH for another 3 min. After rinsing in purified water, sections were coverslipped using aqua poly/mount mounting medium (Polysciences).

### Light microscopy: image acquisition and processing

Stacked brightfield and fluorescence overview images were collected with 10 × , 40 × or 63 × Plan-Apochromat objectives using an automated slide scanner microscope (Zeiss AxioScan Z1) or a fluorescence-phase contrast microscope (Keyence BZ 9000). Stacks were collected at 1–5 µm intervals, merged and post-processed using the corresponding software (ZEN 2.3 or BZ-Analyzer). Shading correction and white balance were carried out. High magnification fluorescence images were obtained with a 63 × Plan-Apochromat (1.2 numerical aperture) objective using a confocal laser scanning microscope (Zeiss LSM 510 Meta or LSM 880 fast Airyscan) with lasers excitation at 543 and 633 nm and emission was detected using a BP 565–615 and a BP 650–710 filter.

#### Quantification of neuronal loss in the substantia nigra [stereological analysis]

The staging of PD according to Braak [11] and the compartmental organisation of the SN into matrix and nigrosomes according to Damier [17, 18] were the neuroanatomical basis of the experimental analysis. All PD cases could be assigned to Braak stages 3 to 6 with two PD brains characterized by an almost complete loss of dopaminergic neurons. In all Parkinsonian brains, highest neuronal cell loss was consistently observed within nigrosome 1.

Stereological analysis was performed using the optical fractionator method according to West et al. [92] as described by Morawski et al. [62]. Numerical densities of TH-positive neurons were determined using a Zeiss (Jena, Germany) Axioskop 2 plus microscope equipped with a motorised stage (Märzhäuser, Wetzlar, Germany), a Ludl MAC 5000 (LEP, Hawthorne, NY, USA) and a digital camera CX 9000 (MicroBrightField, Williston, VT, USA). Stereo Investigator software 7 (MicroBrightField, Williston, VT, USA) was used to analyse serial Sects. (10–12 µm thick) cut frontally through the midbrain encompassing the SN on the level of red nucleus, oculomotor nucleus and superior colliculus. A final section thickness of 10 ± 2 µm was received on average. This permitted for a dissector height of 10 µm using a guard zone of 2 µm on either side of the section. The contour of the SN/nigrosome 1 was outlined in a Stereo Investigator programme using a 5 × lens and the number of cells marked with the primary antibody was counted using an oil-immersion 63 × lens (1.4 numerical aperture). All neurons explicitly stained with at least fifty percent of their soma and one dendritic branch visible within the counting frame were considered. Neurons, which did not fulfil these criteria, were excluded. The coefficient of error (CE) was estimated with a one-stage systematic sampling procedure that has been described by Schaeffer et al. [80]. In parallel, the total number of neurons was determined on Nissl-stained sections by counting all neurons with visible nucleus. All cell counting and density measurements were performed blind to diagnosis by one rater. Statistical analysis by F-test and Student’s t-test was performed on absolute cell numbers (p < 0.05 significance threshold).

## Quantitative iron mapping by immuno-µPIXE elemental analysis

### Tissue preparation

**Fig. 1:**
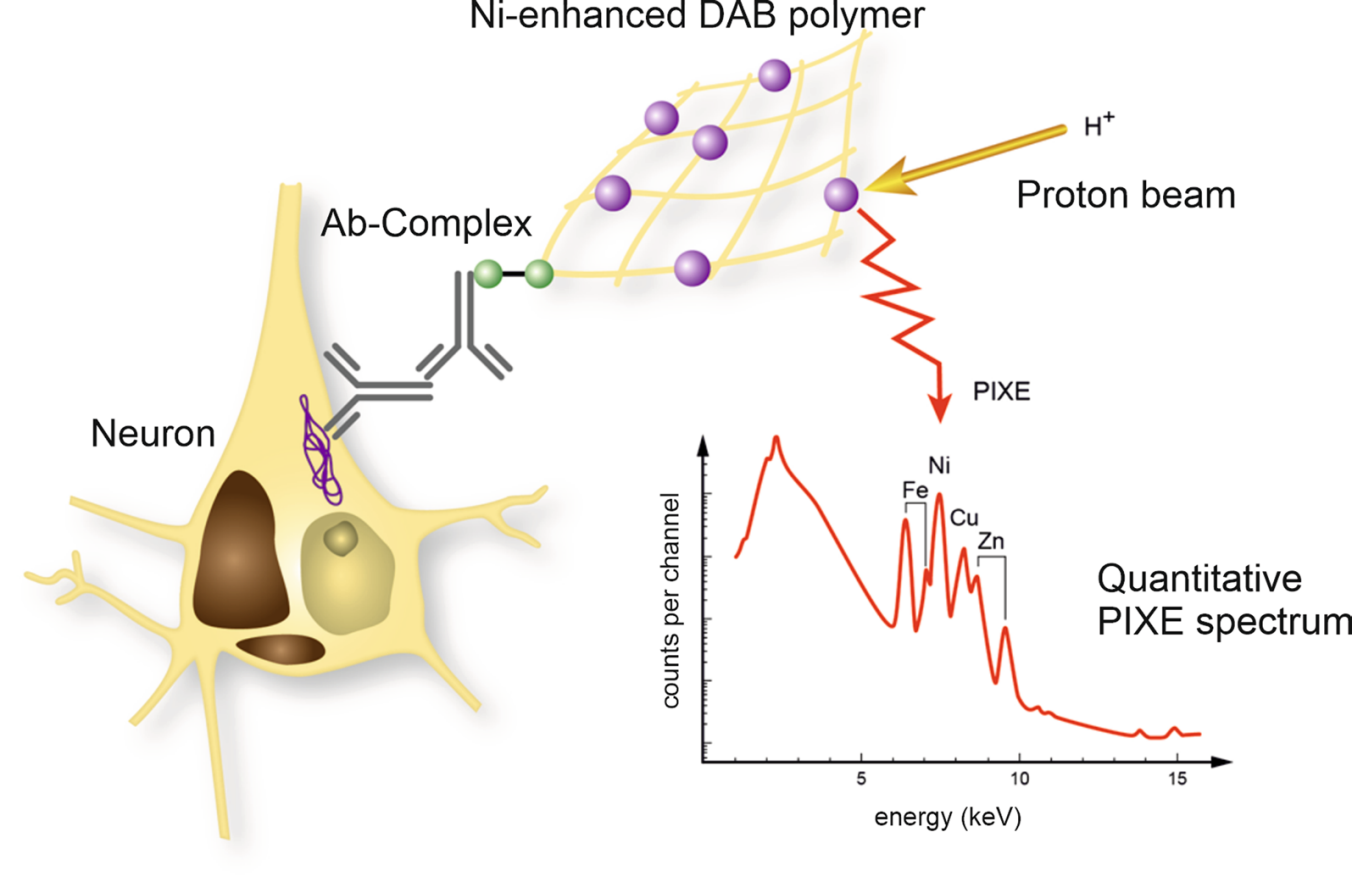
Sample preparation for proton beam analysis. The antibody (Ab)-complex, bound to a cell-type-specific antigen is enhanced with high-purity nickel (violet spheres). Since nickel is specifically identifiable in the PIXE map, it can be used as a marker for any antigen of interest. This combination of antigen labelling with subsequent nickel amplification allows for the application of quantitative PIXE analysis to any immunocytochemical preparation

Sections were treated following the immunohistochemical protocol for nickel-enhanced colour reaction of the target structures with the exception that all buffers and reagents were prepared with ultrapure and fresh substances (to avoid undesired signals in µPIXE), filtered using a pressure filtration system (Sarstedt) with a cellulose acetate membrane without surface-active agents and a pore size of 0.2 µm to prevent iron contamination. The colour reaction was performed using DAB and nickel (99.999% purity grade nickel ammonium sulphate) in Tris–HCl (pH 8.0) (Fig. 1). Sections were dehydrated and incubated overnight in xylol, followed by embedding in a thin layer of mounting medium DePeX (Serva Electrophoresis). After 4 h of drying at room temperature, an area of 15 × 20 mm^2^ was selected, cut out and pulled off the object slide for mounting on an aluminium frame using double sided adhesive carbon tape (G3939, Plano GmbH, Germany). Brightfield microscopy (Keyence BZ 9000) was performed before the µPIXE analysis for orientation, selection and documentation of the regions to be examined.

### Immuno-*µ*PIXE quantitative element mapping

Quantitative element mapping using µPIXE was performed at the ion beam laboratory LIPSION at the Leipzig University (Germany) [75]. The proton induced X-rays were recorded with an energy dispersive high purity germanium detector (Canberra GUL0110) that allows simultaneous detection of characteristic X-rays from all elements above atomic number 11. Since the beam position during the scan is known, quantitative element maps can be projected from the data. The quantification of absolute element concentrations is based on the dynamic analysis method which was implemented in the GeoPIXE analysis software package [79]. It allows spectral deconvolution by fitting element peaks and background, yield calculation from fundamental parameters for X-ray production, matrix effects, geometric parameters and efficiency of the detector. While production cross sections and absorption coefficients for X-rays are precisely known from atomic physics, the detector geometry and efficiency are set-up-specific and need to be determined. The geometric parameters are known from the detector data sheet and from the installation geometry. The detector efficiency which depends on the X-ray energy, is fully described by a model function, based on the underlaying physical processes and their parameters. For the employed X-ray detector, the parameters were calibrated by measuring a set of elements with known concentrations within certified reference materials (SPEC25-53+FC, Astimex Scientific Ltd., Toronto, Canada).

**Fig. 2:**
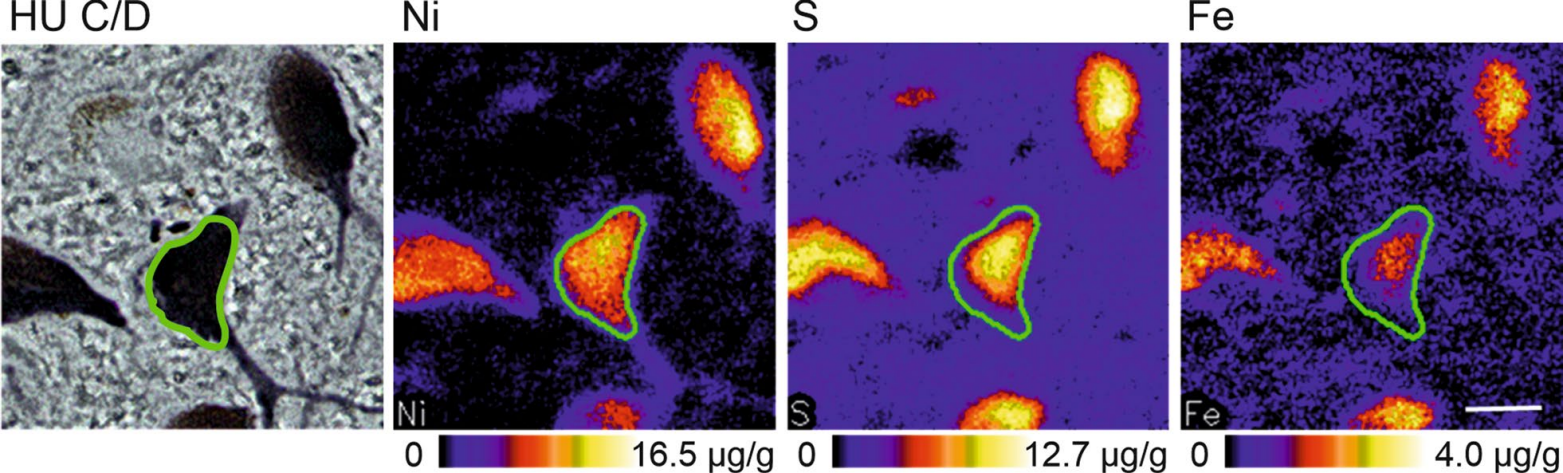
Selection of regions of interest for cellular iron quantification. In the nickel (Ni) map of the immunocytochemical preparation of Hu C/D, the region of interest (ROI) is defined by a spline (in green) marking the cell shape. This ROI applies also to the other element maps from which the concentrations can be extracted. HU C/D: neuronal marker, S: sulphur, Fe: iron. Scale bar 20 μm, applies to all images

The tissue sections were scanned at 2 µm resolution using a focused 2.25 MeV proton beam at a current between 200 and 800 pA for about 3 h per scan. Cellular iron concentrations were extracted by one rater by encircling the cells manually as a region of interest (Fig. 2) using the wide range of graphical tools in GeoPIXE. The detection limit for iron in the cellular regions was about 20 µmol/l [63].

In order to verify that samples were not contaminated with iron due to sample preparation and staining procedure, additional sections were deparaffinised and rehydrated, again dehydrated and embedded in DePeX and analysed. The iron concentrations did not differ significantly between stained and unstained sections.

### MRI

MRI measurements of human midbrain on two samples (one Co and one PD patient from the same pool) were carried out on a whole body 7 T MRI scanner (Magnetom, Siemens, Erlangen, Germany) using a custom-made 2-channel radio-frequency (RF) transmit-receive coil. The brainstem was fixed for six weeks in phosphate buffered 4% formaldehyde solution (pH 7.4). One week prior to MR scanning, the brainstem was transferred into 0.1 M phosphate buffer solution (PBS, pH 7.4) to washout any formaldehyde from the tissue. For scanning procedures, the brain tissue was transferred into fomblin (Fomblin® Y, Sigma-Aldrich). High-resolution quantitative maps of effective transverse (R2*) relaxation rates were obtained using a 3D multi-echo fast low-angle shot (FLASH) acquisition with isotropic resolution of 230 μm, matrix size of 128 × 128 × 60, and five equidistant echo times with bipolar readout and TE1-TE5 = 7.81 ms, 18.11 ms, 27.76 ms, 37.41 ms, 47.06 ms, repetition time TR = 60 ms, flip angle of 27° and bandwidth of 344 Hz/Px Quantitative R2* maps were obtained by a mono-exponential fit of the MR signal decay across different echo times (TEs).

### Statistical analysis

Statistical analysis was performed using the scientific graphic and statistic software SigmaPlot (version 11.0) of Systat Software Inc. (San Jose, CA, USA), and values with p < 0.05 were regarded as statistically significant (Group differences in the Mann–Whitney rank sum test). All values are given as the median [1st quartile, 3rd quartile]. Iron concentrations were compared between controls (Co) and Parkinsonian subjects (PD) and analysed for neurons (Co n = 175; PD n = 111), microglial cells (Co n = 62; PD n = 109), astroglial cells (Co n = 88; PD n = 142), oligodendroglial cells (Olig2: Co n = 44; PD n = 79), ferritin-rich domains within oligodendroglial cells (Co n = 70; PD n = 87) and neuromelanin (Co n = 37; PD n = 30). Group differences were assessed by a Mann–Whitney rank sum test. Significant differences between two groups are marked as follows: * p < 0.05; ** p < 0.005; *** p < 0.001.

## Results

### Stereological quantification of cell loss in the substantia nigra

Neuronal loss in the SN of patients with PD was stereologically quantified on Nissl-stained sections as well as on immunocytochemical preparations for tyrosine hydroxylase (TH), detecting dopaminergic neurons (Table 2).

**Fig. 3:**
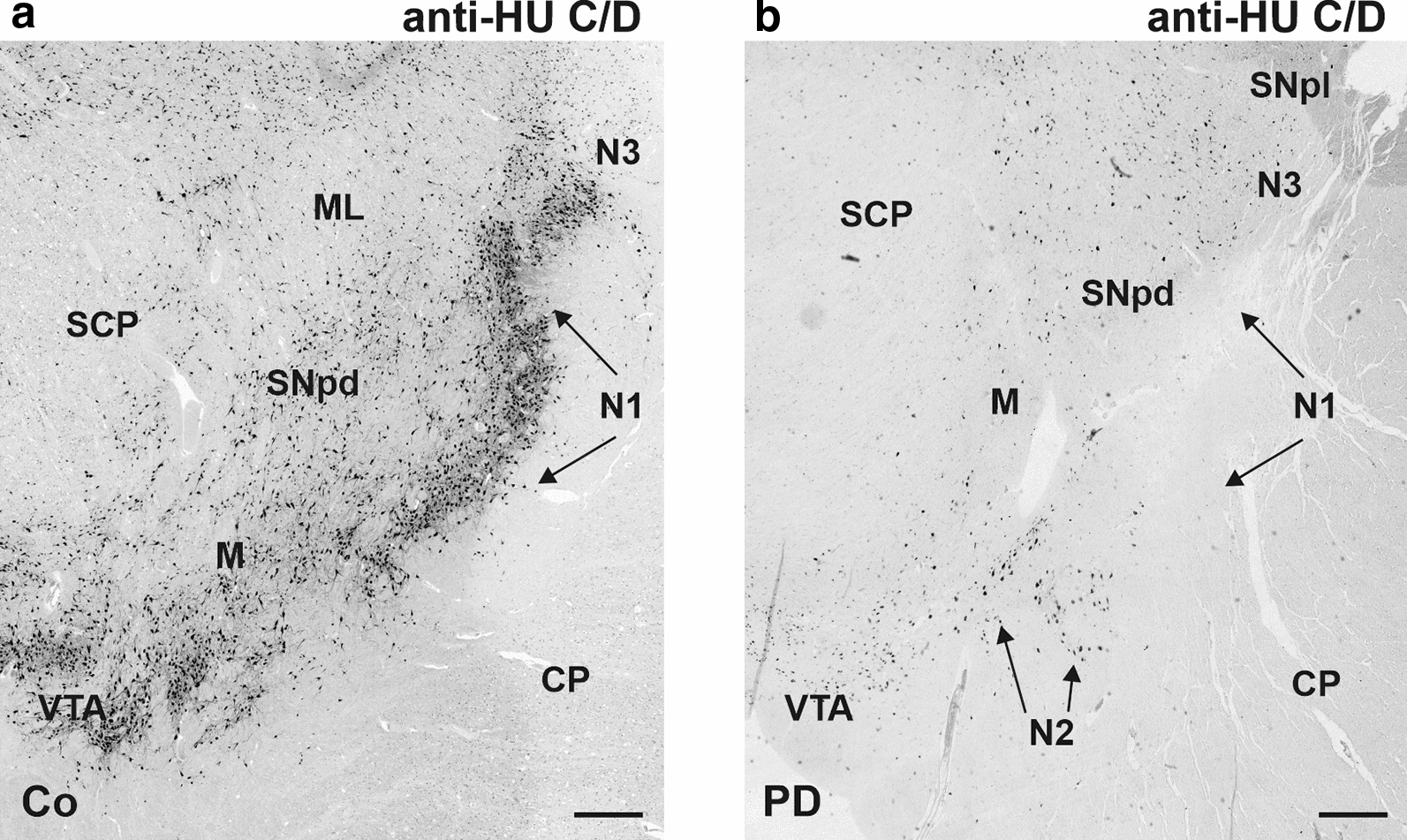
Neuronal distribution in the substantia nigra in a control (Co, left) and a patient with PD (right) immunohistochemically detected by an anti-Hu C/D antibody. Note the most pronounced loss of neurons in nigrosome 1 (N1) CP: cerebral peduncle, M: matrix, ML: medial lemniscus, N: nigrosome, SNpd: dorsal part of SN, SNpl: lateral part of SN, VTA: ventral tegmental area, SCP: superior cerebellar peduncle. Scale bar 500 µm

In controls, the SN appeared as a region in the midbrain with high neuronal density. Out of a total of eight cases with PD, we identified three cases with severe [80–95%] neuronal loss, another three cases with moderate neuronal loss (70–80%) and two cases with a mild neuronal loss [50–60%]. Throughout the SN, loss of neurons was most pronounced in nigrosome 1 in all cases with PD [20, 29] (Fig. 3). Thus, we selected nigrosome 1 as defined by Damier [19, 20] for the subsequent more in-depth analysis.

### Macro-imaging of iron distribution in the substantia nigra with MRI and histochemistry

**Fig. 4:**
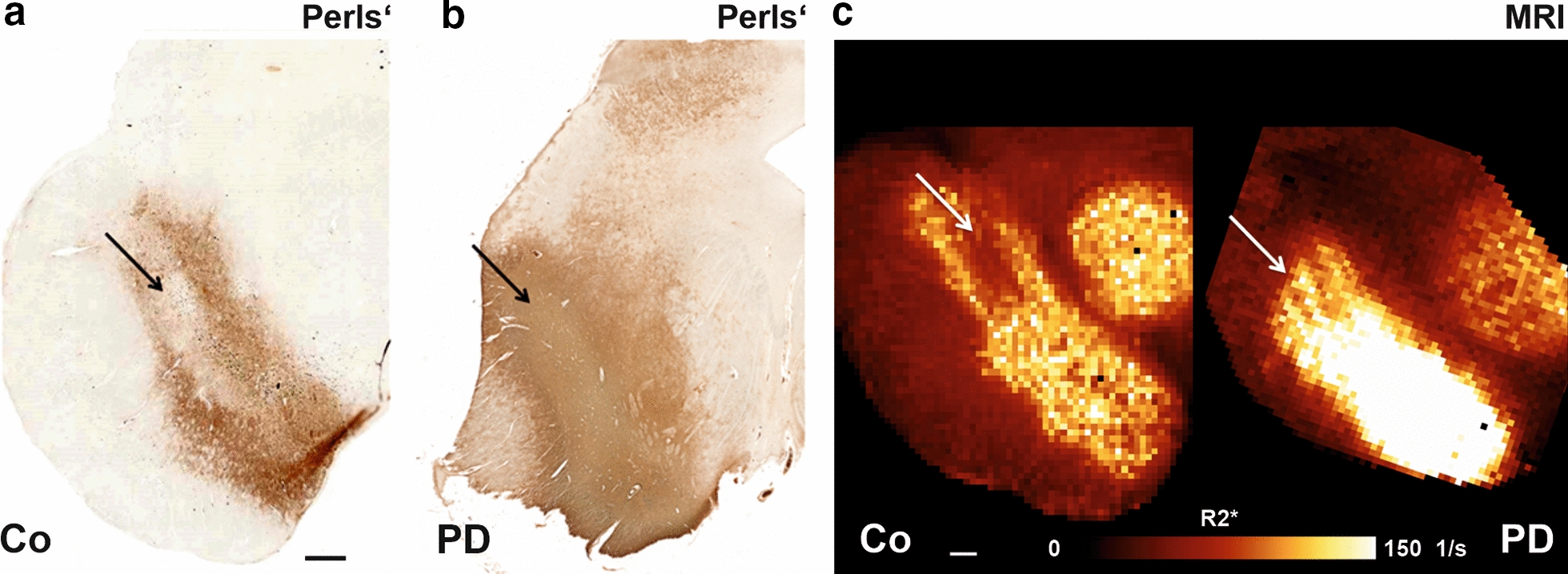
Macroscopic distribution of iron in the substantia nigra obtained by Perls ‘ staining and R2* mapping (MRI). In controls (Co), the area near nigrosome 1 can be delineated by a comma-like hypodense signal (arrows) in both Perls’ staining (A) and MRI R2* maps (C), surrounded by two tails of hyperdense signal, giving rise to the typical “swallow tail” structure. This “swallow tail” pattern disappeared in PD (B, C) because the iron is distributed rather homogeneously, where iron distribution obtained by Perls’ staining (B) shows a more homogeneous distribution throughout the SN. Scale bar 1000 µm

To analyse the iron distribution in the SN and its alterations in PD, we first estimated a semi-quantitative map of non-chelated Fe^3+^ using a DAB-amplified Perls’ stain (Fig. 4a, b). We obtained a very dense staining which clearly defined the SN as an area with high iron precipitation against a pale background (Fig. 4a). While overall, colour precipitation within the SN was rather homogeneous, a ‘comma’-like shape hypodense area surrounded by an upper and lower band of hyperdense staining was clearly delineated near nigrosome 1. This typical staining pattern is also clearly visible on R2* maps of non-neurodegenerative control (Fig. 4c), where it has been described as a typical “swallow tail” shaped sign. It is considered as a specific marker to identify healthy substantia nigra unaffected by degenerative changes [50]. In PD, the typical “swallow tail” sign disappears in R2* images (Fig. 4c) and in the Perls’ staining pattern and the distribution of iron becomes more homogeneous throughout the SN (Fig. 4b). The remarkable similarity of the Perls’ iron staining pattern and the MRI R2* map, both forming the “swallow tail” sign, which disappeared in PD, adds additional support for the diagnostic value of this structural feature. This observation points towards increase of overall iron content in SN of PD patients in areas close to nigrosome 1.

### Iron rich cellular structures on Perls’ reaction

**Fig. 5:**
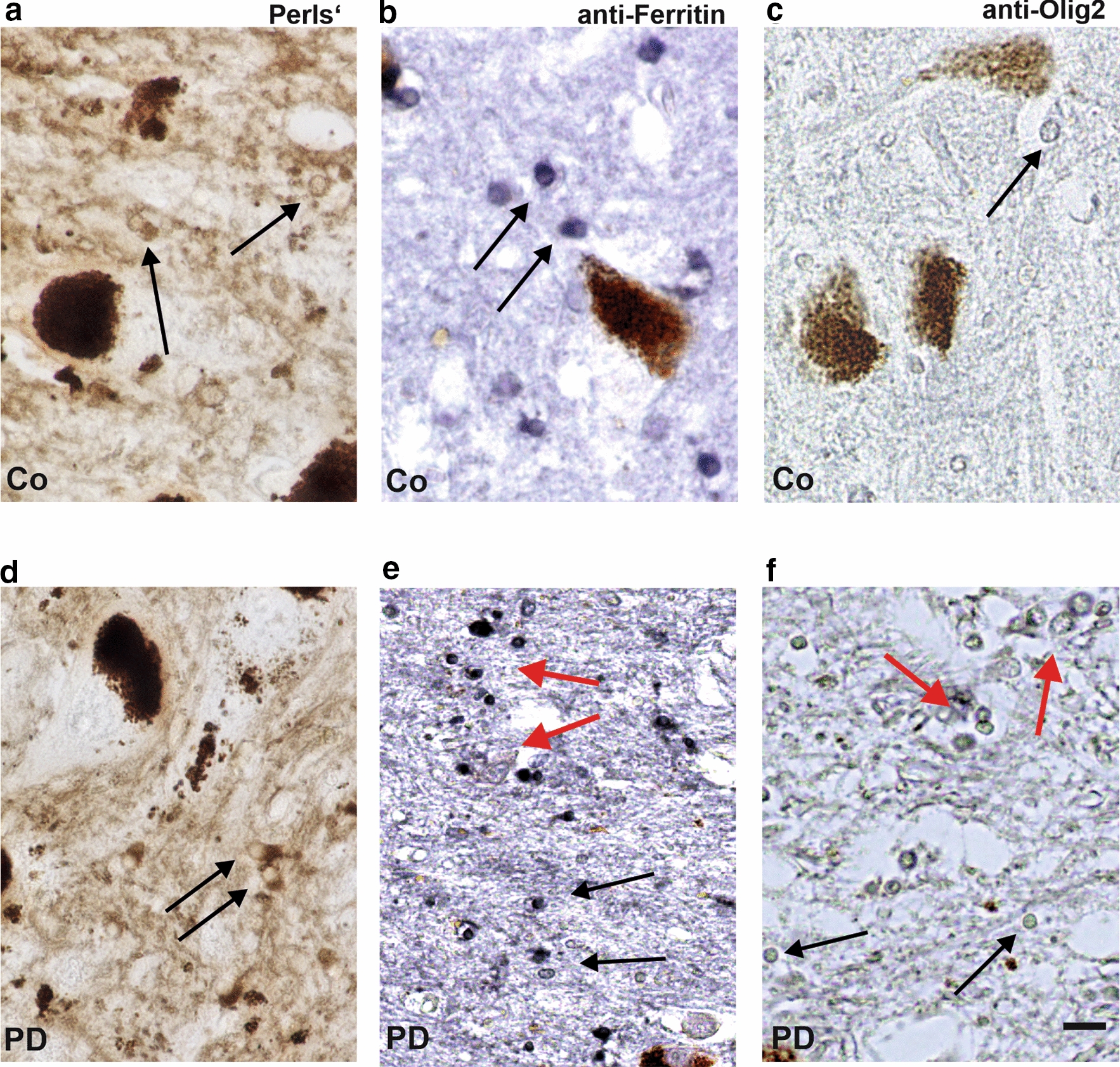
Perls' reaction (**a**, **d**), anti-ferritin- (**b**, **e**) and anti-Olig2- (**c**, **f**) immunoreactivity of oligodendroglia cells (arrows) in human SN nigrosome 1 in controls (Co, **a–c**) and patients with PD (**d–f**). Scale bar 10 µm

A higher magnification of DAB-amplified Perls’ stain allows for identifying cell types that are characterised by a high accumulation of non-chelated Fe^3+^ (Fig. 5a, d). Above all, oligodendroglial cells showed an intense cytosolic Perls’ reaction for non-chelated Fe^3+^ (Fig. 5a, d). The surrounding parenchyma appears inhomogeneously coloured and shows fibre-like morphological characteristics. Oligodendroglial cells detected with an anti-ferritin antibody also showed a higher immunoreactivity in cytosolic regions than in the nucleus (Fig. 5b, e). In PD, oligodendroglial cells (Fig. 5c, f, red arrow) with a positive immunoreaction for ferritin appear tighter in nigrosome 1 supporting reactive changes potentially leading to oligodendrogliosis (Fig. 5b, e).

### Cell-type specific quantitative mapping of iron by immuno-µPIXE

**Fig. 6:**
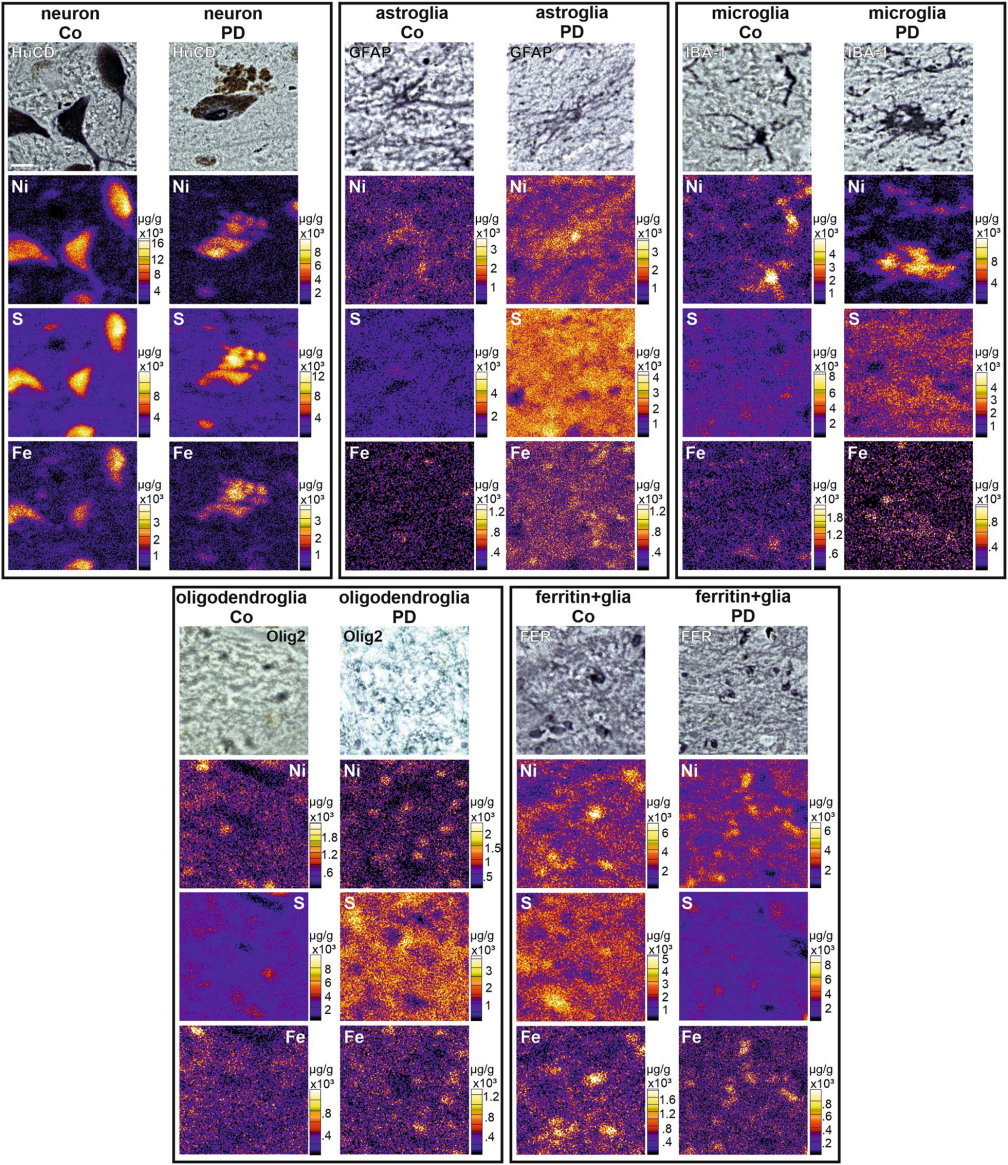
Element maps in human SN nigrosome 1 in controls (Co) and patients with PD, obtained by µPIXE. Brightfield microscope images (top row) show the immunocytochemical reaction for cell-type specific antigens providing the basis for definition of regions of interest for the subsequent PIXE imaging (below). The ultrapure nickel-enhancement of the immunoreaction allows to allocate the µPIXE element signals to specific cell types. The iron map was used to quantify the local concentrations of iron in different cell types (see Fig. 7). Scale bar top left 20 µm applies to all images

In order to bridge the gap between macro and micro-imaging in PD and to further specify the iron distribution and its disease-associated changes, we applied immuno-µPIXE. Typical examples of microscopic brightfield images of immunocytochemical preparations and corresponding µPIXE element maps are shown in Fig. 6.

The distribution of sulphur in the PIXE map largely corresponds to the neuronal pigment neuromelanin, which, due to its iron-sulphur-clusters, is enriched in sulphur (approx. 10 × 10^3^ µg/g). Extracellular sulphur (approx. 2.5 × 10^3^ µg/g) mainly represents chondroitin sulphate proteoglycans (CSPG), the main components of the extracellular matrix. Iron was found to be concentrated in neurons and glial cells, which both showed higher cellular cytosolic iron loads than their extracellular environment (Fig. 6, bottom). Neuronal iron deposits typically co-located with neuromelanin which has two iron binding sides, with high and low affinity for iron, respectively.

**Fig. 7:**
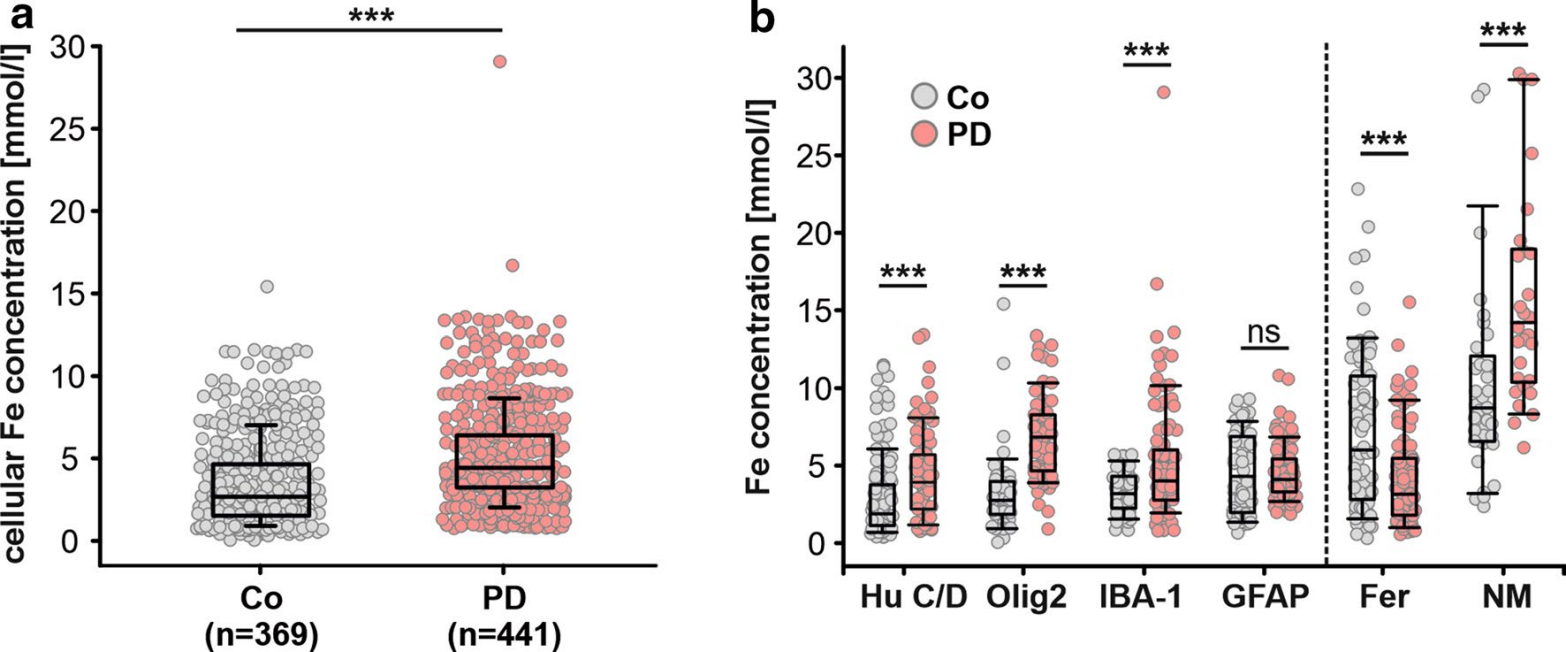
Quantification of iron in nigrosome 1 neurons in controls (Co) and patients with PD (**a**). The cellular compartmentation of iron in the SN nigrosome 1 in controls (Co) and patients with PD for different cells types, ferritin positive cells and in the pigment neuromelanin (**b**). Cellular concentrations of iron were obtained through quantitative element mapping by µPIXE in combination with nickel-enhanced immunocytochemical detection of cell type-specific antigens. Data are given as median values ± SD. n: analyzed cell number; Neurons (Hu C/D), Oligodendrocytes (Olig2), Microglia (IBA-1), Astroglia (GFAP), Ferritin (Fer) and Neuromelanin (NM)

PD, as compared to controls was associated with both changes in the total content of cellular iron and a shift in the cellular distribution of iron (Fig. 7a, b). The average total cellular iron of cells in nigrosome 1 in healthy controls amounts to 2.7 mM [1.5, 4.6] (n = 369) and to 4.3 mM [3.2, 6.4] (n = 441) in PD which corresponds to an increase by about 62% (Fig. 7a). Of note, total cellular iron content showed a very large individual variability which in healthy controls reached from 0.30 mM to 29 mM.

The cellular compartmentalisation of iron in the SN was altered in PD patients and iron upregulation in PD showed differences in the variability for different cell types (Fig. 7b). In anti-Hu C/D reactive neurons, iron concentration was about doubled in PD compared to controls. Glial cells, where iron concentrations in controls tended to be higher than in neurons, showed more complex changes in PD with an increase in most cell types (Fig. 7b).

In oligodendroglial cells, that were subdivided according to the presence of specific antigens into functionally different, i.e. myelinating and non-myelinating (satellite) subtypes, iron concentrations in anti-Olig2-immunoreactive oligodendroglial cells were increased from 2.8 mM [1.9, 4.0] (n = 44) by about 150% up to 6.8 mM [4.7, 8.3] (n = 79). In anti-ferritin-immunoreactive oligodendroglial cells, that showed the highest iron concentration among all cell types in controls, iron concentrations in PD cases dropped to 51% (controls: 6.0 mM [2.8, 10.8] (n = 70); PD: 3.2 mM [1.8, 5.5] (n = 87)). This reduction can be caused by a depletion of ferritin, the main form of iron storage in glial cells, or by a reduction in ferritin loading. Iron concentrations in PD in anti-IBA-1 reactive microglial cells were increased by about one quarter (26%, controls: 3.2 mM [2.2, 4.3] (n = 62); PD: 4.0 mM [2.8, 6.0] (n = 109)) but remained unaffected in anti GFAP reactive astroglial cells (controls: 4.3 mM [2.0, 6.9] (n = 88); PD: 4.1 mM [3.3, 5.4] (n = 142)).

In addition to the specific changes in the cellular compartmentalisation of iron in PD, the iron loading of neuromelanin as a structure with significant iron binding capacity must be considered. Contrary to controls, where neuromelanin is restricted to dopaminergic neurons, it is found in PD predominantly extracellularly which could be due to remains of disintegrated neurons and therefore a consequence of neurodegeneration. The iron concentration of neuromelanin in PD was increased by about two thirds (62%, controls: 8.7 mM [6.6, 12.1] (n = 37); PD: 14.2 mM [10.4, 19.0] (n = 30)).

### Iron storage proteins, oligodendroglial cells and myelin

**Fig. 8:**
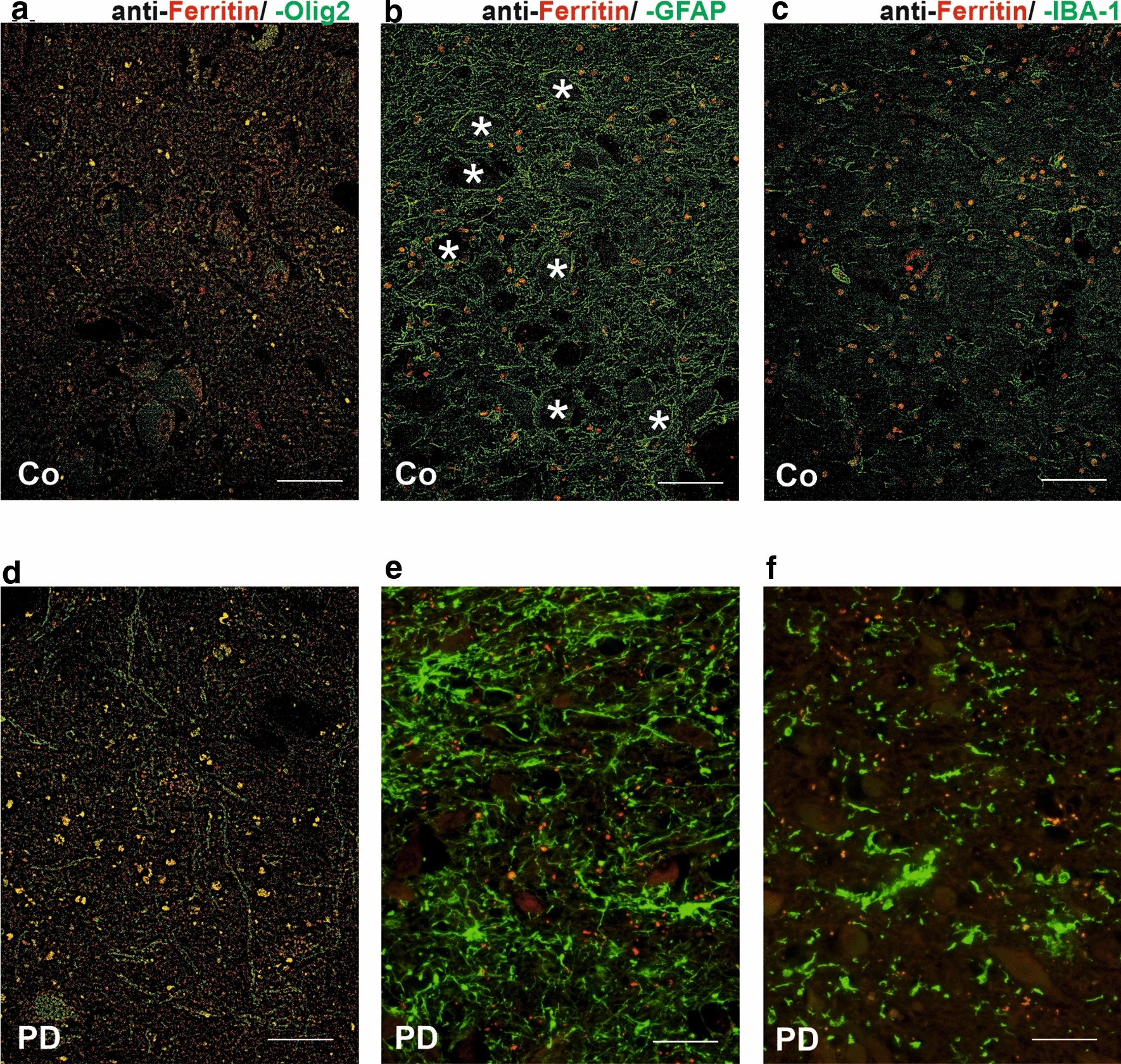
Double-label fluorescent immunohistochemistry with anti-ferritin and anti-glial cell markers in nigrosome 1 in controls (Co, **a–c**) and patients with PD (**d–f**). Anti-ferritin antibody (red) in combination with anti-Olig2 antibody (green) (**a**, **d**), anti-ferritin antibody (red) combined with anti-GFAP antibody (green) (**b**, **e**) and anti-ferritin antibody (red) combined with anti-IBA-1 antibody (green) (**c**, **f**) did not show significant spatial co-localization whereas immunoreactivity in oligodendrocytes (**a**, **d**) could be confirmed. Immunohistochemical observations with anti-ferritin antibody show positive reactions in oligodendrocytes. In addition, (**e**) and (**f**) show strong activation signs of glial cells including a hypertrophic morphology and an increase in cell density. Territories of neurons surrounded by glia cell processes (**a**) and (**d**) are labelled with *. Sections were treated with Sudan black B to suppress tissue auto fluorescence. Scale bar 50 µm

To assign the strong ferritin-associated µPIXE iron signal to defined cellular structures, we performed double-label fluorescent immunohistochemistry with anti-ferritin and anti-glial cell markers. As illustrated in Fig. 8, ferritin immunoreactivity both in controls and PD was largely restricted to oligodendroglial cells (yellow colour overlap, Fig. 8a, d) while astroglial or microglial cells showed no co-immunoreaction (Fig. 8b, e and c, f).

**Fig. 9:**
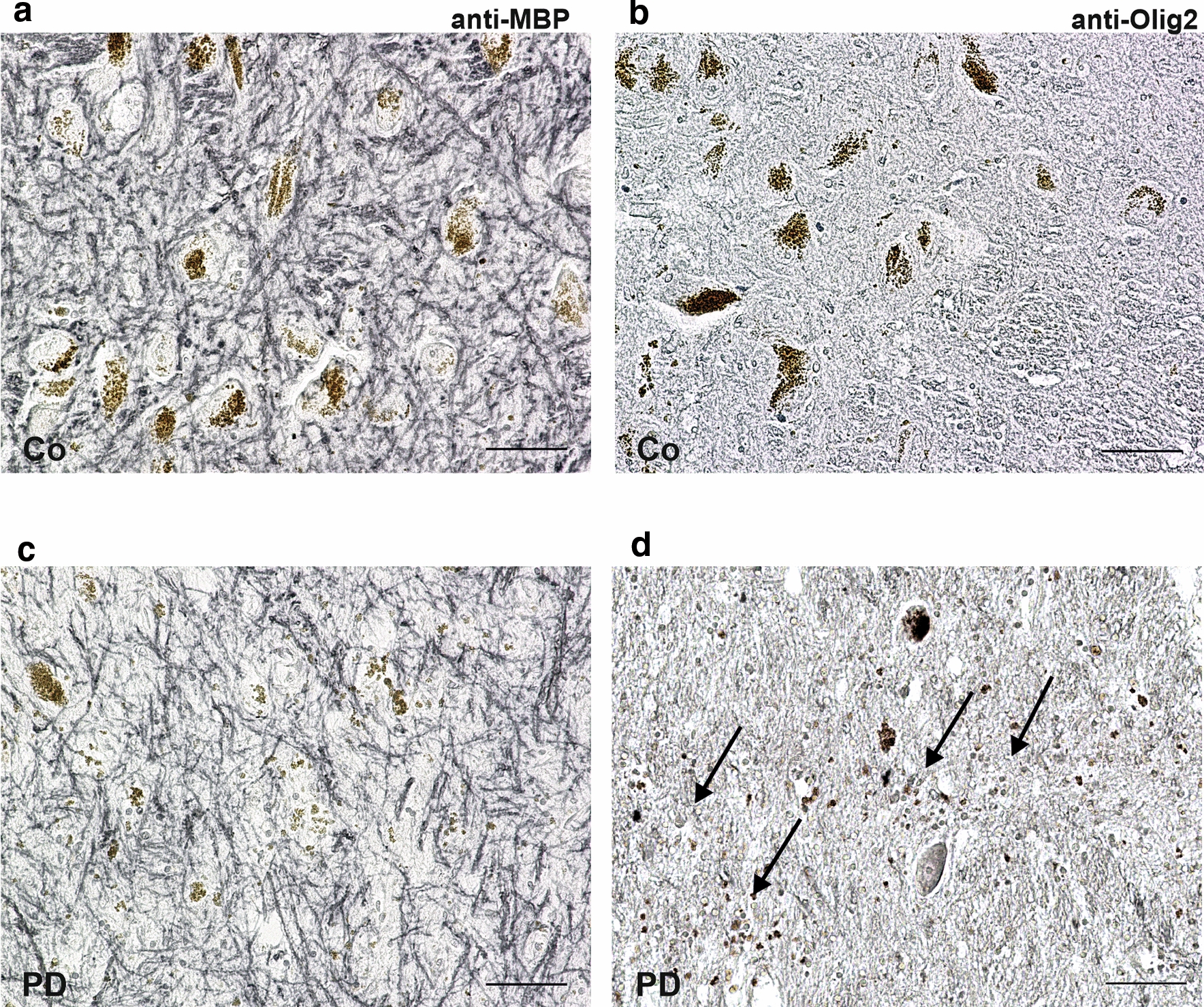
Oligodendroglia activation and degenerative change of myelin in nigrosome 1 of controls (Co, **a**, **b**) and PD (**c**, **d**) patients. Brightfield microscopy of myelin basic protein (MBP) shows a dense and rather homogeneous network of myelinated fibres in controls (**a**). In PD, myelinated fibres appear less dense (**c**). Additionally, in PD an increase of anti-Olig2 positive oligodendrocytes is visible (arrows, **d**). Scale bar 50 µm

Overall, oligodendroglial cells showed the most pronounced changes in iron compartmentalisation in PD. To further characterise pathology related changes in oligodendroglial cells, myelinating and non-myelinating (satellite) subtypes were investigated differentially. To this end, sections were immunolabelled with anti-Olig2 and antibodies against myelin basic protein (MBP) (Fig. 9). Morphological appearance and immunoreactivity for both antigens differed between controls and PD. While myelinated fibres in controls are more homogeneously distributed (Fig. 9a), they appeared rarefied, more delicate and partially agglomerated in PD (Fig. 9c). Sections labelled with an anti-Olig2 antibody show an increase in cell density of stained oligodendroglial cells in PD (Fig. 9d). Observed reactive morphological changes could be due to previous neurodegeneration leading to a secondary degenerative shift of myelin or may show direct evidence of oligodendrogliosis.

## Discussion

Iron is one of the key factors that most likely plays a major role in PD although its precise pathogenic role is not fully understood [98]. While several studies report on increased levels of total iron in the SN [21, 22, 26, 40, 43, 51, 77, 84], others did not [33, 49, 87]. This increase of total nigral iron may reflect an interference of multiple pathological processes including inflammation, myelination, perturbed iron uptake as well as vascular damage, each affecting distinct cell populations. In addition, there are various pre-analytical and analytical factors of iron determination including tissue acervation and sample preparation that may account for such discrepancies.

An incubation of brain tissue in formalin for four years was shown to decrease iron levels down to 57% compared with similar analysed fresh frozen tissue [81]. It is reasonable to assume that iron leaching depends primarily on the fixation time, chemical form and to a minor extend on the pathology. Thus, the relative increase or decrease in iron concentrations of PD brains compared to control brains is only marginally affected in our study. Different or larger extends of iron leaching are not expected since the sample preparation processes followed the same protocol with fixation times less than half a year. To test the extend of preparatory effects, we have analysed the iron concentrations of nine neuromelanin deposits in three sections of a cryofixed and unstained substantia nigra from control brain. The average iron concentration of neuromelanin from the cryofixed tissue was 8.0 mM [6.4, 11.2] (n = 9), which is in agreement with the results of 8.7 mM [6.6, 12.1] (n = 37) for neuromelanin of short-term paraformaldehyde-fixed substantia nigra from control brains.

Semi-quantitative histochemical methods were able to demonstrate that iron deposits were present in both neurons and glial cells of the SN, with an increase of ferritin-loaded microglial cells in the SN [43]. Still, precise quantitative data on cellular iron compartmentalisation in the brain and its changes in PD are still lacking.

Here, we close this gap in knowledge by applying immuno-µPIXE to the human brain, a newly developed technique which allows a quantitative determination of major and trace elements and their assignment to specific cell types. In particular it is suitable to analyse iron in brain sections with a spatial resolution in the µm-range and a detection limit of approximately 10 µM [31, 57, 59–61, 73, 74, 76].

We determined a tissue concentration of total iron in nigrosome 1 in controls of 2.7 mM [1.5, 4.6] (n = 369) and of 4.3 mM [3.2, 6.4] (n = 441) in PD. To convert this for comparison into ng/mg wet tissue weight, the values amount to 180 ng/mg in control and of 300 ng/mg in PD. This in in good agreement with values reported by Friedman et al. [32], Dexter et al. [22] and Riederer et al. [77].

In PD, our results show an increase of total iron content within the nigrosome 1 as compared to controls by about two thirds (62%) which is in line with previous investigations that reported an increase in total iron in PD [23, 36, 71, 84]. However, we for the first time demonstrate that the increase of total iron differs across cell types in PD.

Most strikingly was the twofold increase (207%) of cellular iron within the dopaminergic neurons of nigrosome 1 in PD, which is in line with previous reports [1, 60, 65, 66]. This finding may provide an insight in the pathological role of iron accumulation in PD. Due to the rather unaffected sulphur content, our results confirm that neuromelanin is unsaturated with an Fe/S ratio of 10 at% under physiological conditions. A similar physiological Fe/S ratio of 13 at% was obtained with high resolution analysis directly on single neuromelanin granules [7]. Sulphur in the granules originate from pheomelanin, which derives from 5-S-cysteinyldopamin. Structurally, pheomelanin seem to favour the formation of the low-affinity mononuclear iron centre [30]. Eumelanin, the other melanin moiety, forms the high-affinity site where iron remains redox-inactive. Thus, neuromelanin has physiologically a protective function, since iron, bound preferentially to its high-affinity binding sides, is segregated from supporting any redox processes [96, 102]. Under conditions of increasing iron overload, which may result from alterations in iron metabolism [5, 6, 45, 78], high-affinity eumelanin becomes saturated and the low-affinity binding sites of pheomelanin are increasingly occupied. Then loosely bound iron can be released easily and trigger further toxic reactions that may lead to progressive neurodegeneration [28]. A pathogenic role of elevated iron concentrations is also supported by reports of direct correlations between the severity of neuronal loss in PD and the amount of iron accumulation [52, 93].

However, despite the high increase in neuronal iron concentration, it only contributes little to the overall increase of iron in nigrosome 1. Of note, both in the normal brain and in PD, the major iron pool (about 80%) is localised in glial cells. In the normal brain, the population of ferritin-stained oligodendroglial cells contain most iron, followed by astroglial cells, microglial cells and another population of Olig2-stained oligodendroglial cells (ferritin-stained oligodendroglial cells > astroglial cells > microglial cells > Olig2-stained oligodendroglial cells). This pattern is inverted in PD (Olig2-stained oligodendroglial cells > microglial cells > astroglial cells > ferritin-stained oligodendroglial cells). While the iron content of astroglial cells in PD remained unchanged, microglial cells showed an iron accumulation of about one quarter (26%) and Olig2-stained oligodendroglial cells by about 150%. Descriptions of the physiological and pathophysiological role of iron need to take into account the iron redistribution between neurons and glial cells.

The increase of iron in microglial cells has been attributed to its activation [6, 86, 99] and might be due to phagocytosis of neuromelanin fragments [94, 97, 100, 102] and increased iron uptake via transferrin-receptor [91] or non-transferrin bound iron [8]. Accordingly, to our morphological observations, microglial cells have the potential to degrade and solubilize neuromelanin that is released by degenerating neurons [100]. As a result of this, soluble iron is generated that could be internalized in other surrounding cells, promoting neuroinflammation and ultimately neurodegeneration. Extracellular neuromelanin goes hand in hand with a release of proinflammatory and toxic molecules that can maintain neuroinflammation as well as degeneration processes [100]. The iron pool of activated microglial cells may again be subject to cellular redistribution. Apoptosis of exhausted and damaged microglial cells might result in release of iron into the extracellular space [12] and circulating toxic iron may be taken by neurons or glial cells through transferrin-dependent and independent mechanisms [5, 8, 78, 86]. Activation of microglial cells, furthermore, is associated by a reduction of ferritin-*de-novo*-synthesis and H-ferritin levels, which may occur as a consequence of increased oxidative stress [55, 56]. Although the concentration of L-ferritin in the SN is lower than that of H-ferritin, levels of L-ferritin increased as well as H-ferritin levels during the normal ageing process [98]. Instead, in PD, decreasing levels of H-ferritin are described by Zecca et al. [98]. In accordance to these investigations, we equally observed a reduction of ferritin deposits in glial cells in PD down to nearly 50%.

Strikingly, changes of the iron content in PD were most pronounced in oligodendroglial cells. Oligodendroglial cells may hold one of the highest oxidative metabolism of all brain cells [64] and may thus be particularly vulnerable to sequelae of oxidative stress. Due to their involvement in myelin synthesis, oligodendroglial cells may show high concentrations of cellular iron and ferritin [14, 15, 66]. Since oxidative stress in microglial cells contributes to a decrease in H-ferritin levels [55, 56], there is reason to suppose that cellular stress may be reflected as well in a reduction of H-ferritin in oligodendroglial cells. The reduction of both neuromelanin-bound iron and ferritin-bound iron might result in an increase of the labile iron pool, which will further promote oxidative damage.

Astroglial cells even in the normal brain contain a comparatively large cellular iron pool that may play a role in neurotransmitter homeostasis [90]. Astrocytic processes form parts of the blood brain barrier [4, 25] and regulate the uptake and distribution of metal ions within the brain [24, 41, 67, 89, 90], which makes them the first parenchymal cell type that will get in contact with iron after having crossed the blood brain barrier [41]. Due to their large intracellular iron pool together with their close contacts to various other brain cell types [3, 35, 39, 67, 89], they play a key role in processes of iron distribution within the neuropil [68]. The cellular iron concentration in astrocytes remains unchanged in PD, which may indicate that they are largely able to maintain their homeostatic pathways, even under inflammatory conditions.

Taken together, we demonstrated that the total increase of iron concentration in PD is a result of multiple physiological processes differentially affecting different cell populations.

Since iron is involved in both physiological and pathophysiological processes, in order to identify potentially modifiable therapeutic targets, it is essential to accurately determine its cellular compartmentalisation, intercellular re-distribution or differential accumulation both under normal and diseased conditions. While, the pathogenic role of elevated iron in PD remains unquestionable in general, more in-depth knowledge on iron pools and iron fluxes is still lacking. Our immuno-µPIXE approach allows for a highly sensitive quantification of iron at the subcellular level in well preserved cellular architectures. We believe that this knowledge will eventually contribute to the development of disease modifying strategies.

## Data Availability

The data that support the findings of this study are available from the corresponding author M.M. upon reasonable request.
